# Role of Nociceptin/Orphanin FQ and NOP Receptors in the Response to Acute and Repeated Restraint Stress in Rats

**DOI:** 10.1111/j.1365-2826.2012.02361.x

**Published:** 2012-11-20

**Authors:** G Delaney, K L Dawe, R Hogan, T Hunjan, J Roper, G Hazell, S J Lolait, A J Fulford

**Affiliations:** * Centre for Comparative and Clinical Anatomy, University of BristolBristol, UK; † Henry Wellcome Laboratories for Integrative Neuroscience and Endocrinology, University of BristolBristol, UK

**Keywords:** ACTH, glucocorticoids cortisol/corticosterone, neuropeptides, nociceptin, mRNA expression, restraint

## Abstract

Central nociceptin/orphanin FQ (N/OFQ)-expressing neurones are abundantly expressed in the hypothalamus and limbic system and are implicated in the regulation of activity of the hypothalamic-pituitary-adrenal axis (HPA) and stress responses. We investigated the role of the endogenous N/OFQ receptor (NOP) system using the nonpeptidic NOP antagonist, JTC-801 [*N*-(4-amino-2-methylquinolin-6-yl)-2-(4-ethylphenoxy-methyl)benzamide monohydrochloride], during the HPA axis response to acute physical/psychological stress (60 min of restraint). Although i.v. JTC-801 (0.05 mg/kg in 100 μl) had no significant effect on restraint-induced plasma corticosterone release at 30 or 60 min post-injection, i.v. JTC-801 (0.05 mg/kg in 100 μl) in quiescent rats significantly increased basal plasma corticosterone at the 30-min time-point compared to i.v. vehicle (1% dimethysulphoxide in sterile saline). Central injection of JTC-801 i.c.v. was associated with increased Fos expression in the parvocellular paraventricular nucleus 90 min after infusion compared to vehicle control. These findings contrast to the effects of i.c.v. UFP-101, a NOP antagonist that we have previously shown to have no effect on HPA activity in quiescent rats. To determine whether restraint stress was associated with compensatory changes in N/OFQ precursor (ppN/OFQ) or NOP receptor mRNAs, in a separate study, we undertook reverse transcriptase-polymerase chain reaction and *in situ* hybridisation analysis of ppN/OFQ and NOP transcripts in the brains of male Sprague–Dawley rats. In support of an endogenous role for central N/OFQ in psychological stress, we found that acute restraint significantly decreased preproN/OFQ transcript expression in the hippocampus 2 h after stress compared to unstressed controls. PpN/OFQ mRNA was also reduced in the mediodorsal forebrain 4 h after stress. NOP mRNA was reduced in the hypothalamus 2 h after restraint and at 4 h in mediodorsal forebrain and hippocampus. *In situ* hybridisation analysis showed that acute restraint significantly decreased ppNN/OFQ in the central amygdala, with significantly increased expression in bed nucleus and reticular thalamus associated with repeated restraint. There was a strong trend for reduced NOP mRNA in the bed nucleus of acute and repeated restraint groups, although there were no other significant changes seen. Although the exact mechanisms require elucidation, the findings obtained in the present study provide evidence indicating that the endogenous N/OFQ system is involved in both acute and chronic restraint stress responses. In summary, our findings confirm the significant role of endogenous NOP receptors and tonic N/OFQ function in the response to the psychological stress of restraint.

Nociceptin/orphanin FQ (N/OFQ) is a mammalian neuropeptide that exerts diverse actions in the central nervous system and is implicated in the neurobiological control of stress and associated adaptive behaviours. Studies of N/OFQ peptide function have adopted pharmacological and molecular biological approaches aiming to address the involvement of the endogenous peptide and its NOP receptor in stress adaptation. Recent work by ourselves and others involving central infusions of N/OFQ peptide or the competitive, peptidic NOP antagonist, UFP-101, have suggested that acute stress-induced hypothalamic-pituitary adrenal (HPA) axis activity is dependent upon activation of NOP receptors and increased release of N/OFQ peptide (1–4). To further explain the endogenous functions of the N/OFQ system, studies involving exogenous application of selective, long-acting, nonpeptidic NOP antagonists are necessary to determine the role of NOP receptors in basal and psychological stress-induced HPA axis activity. We hypothesise that NOP receptors provide tonic regulation to the HPA axis under conditions of psychological stress. Previous studies have shown that the 4-aminoquinoline derivative, JTC-801 [*N*-(4-amino-2-methylquinolin-6-yl)-2-(4-ethylphenoxy-methyl)benzamide monohydrochloride], inhibits N/OFQ ligand binding, and also antagonises N/OFQ-induced suppression of forskolin-enhanced cAMP accumulation in NOP-expressing human cell lines (5) and the rat cerebral cortex *in vitro* in a dose-dependent manner (6). JTC-801 also attenuates N/OFQ-induced suppression of [^3^H]-5-HT and noradrenaline release in the rat brain, showing time-dependent profiles (6). JTC-801 is relatively nonpolar and appears to have good bioavailability, readily crossing the blood–brain barrier (5), and has been suggested to lack partial agonist activity (7).

JTC-801 has been made widely available commercially and has been employed in *in vivo* physiological research. JTC-801 exerts anti-nociceptive actions in acute pain models when given i.v. or orally (5,8). JTC-801 dose-dependently blocks tactile allodynia induced by L5/L6 spinal nerve injury when given systemically or intrathecally (9). In addition, JTC-801 attenuates thermal hyperalgesia in mice when given orally (10) and N/OFQ-induced thermal hyperalgesia when given intrathecally (11). This NOP antagonist also attenuates formalin-induced hyperalgesia (9) and cannabinoid-induced hypothermia (12). Because JTC-801 is able to inhibit pain behaviours with similar response profiles over a range of doses following administration by various routes, it follows that JTC-801 must readily pass the blood–brain barrier to exert actions in the central nervous system. This would be consistent with the small molecular nature of JTC-801 and its lipid solubility (5). However, aside from pain and thermomodulation, the compound has not been studied with respect of other physiological states or behaviours. The present study aimed to validate our past findings using UFP-101 and to investigate the actions of an additional putative NOP antagonist *in vivo* with respect to endogenous tonic regulation of the stress response by NOP receptors. Because the peptidic NOP antagonist UFP-101 influences the magnitude of the HPA axis stress response in rats (3,4), we administered JTC-801 aiming to examine its effect on restraint stress-induced HPA axis responses. The specific mechanisms responsible for the effect of restraint in rats are incompletely understood and we hypothesise that N/OFQ has a neuromodulatory role in stress, whereby alteration of the expression of endogenous N/OFQ peptide and its NOP receptor mRNA levels are important for adaptation to restraint stress. Because the promoters for both N/OFQ precursor and NOP receptor genes express putative glucocorticoid receptor regulatory elements, it is entirely possible that the transcription of both genes is regulated by stress and associated fluctuations in glucocorticoid hormones. We have previously reported immune stress-induced changes in transcript expression in the rat forebrain using the reverse transcriptase-polymerase chain reaction (RT-PCR) (4); therefore, to demonstrate how restraint regulates site-specific changes in endogenous N/OFQergic system in the limbic forebrain, we undertook RT-PCR analysis and *in situ* hybridisation histochemistry (ISHH) to monitor temporal changes in N/OFQ precursor (ppN/OFQ) and NOP mRNA transcript expression in restraint-stressed rats. We investigated acute changes in mRNA expression to coincide with the expected response to HPA axis activation following restraint (mRNAs at 2–4 h post stress onset). The response of N/OFQ gene transcripts to single or repeated restraint stress was also assessed using ISHH for improved spatial expression analysis. These collective studies provide important new information on the action of JTC-801 on basal HPA axis activity in rats, as well as the neuroanatomical basis of adaptive changes in the endogenous N/OFQ system following restraint.

## Materials and methods

### Animals

Adult male Sprague–Dawley rats weighing 200–250 g (Harlan Laboratories, Blackthorn, UK) were housed in a temperature and humidity controlled environment under a 12 : 12 h light/dark cycle. Food and water was available *ad lib*. and all experiments were carried out in strict accordance with UK Home Office regulations [Animals (Scientific Procedures) Act 1986] and approved by the appropriate University of Bristol Ethical Review Group.

### Restraint stress protocol

Stress procedures were conducted during the circadian nadir (i.e. between 09.00 h and 10.00 h). Rats undergoing acute stress were subjected to 60 min of restraint in ventilated, closed plastic tubes that allowed only limited lateral movement. This model has been validated to elicit a physical/psychogenic stress response associated with stimulation of the HPA axis (3). For mRNA analysis studies by RT-PCR, rats were killed 2 or 4 h after the onset of restraint. For all restraint experiments, control subjects remained in their homecage throughout the duration of the experiment.

### Surgery

Three days before Experiment 1, rats were implanted with an i.v. cannula placed in the right external jugular vein under Saffan anaesthesia (1.4 ml/kg; Schering-Plough, Welwyn Garden City, UK). Following surgery, rats were individually housed. During the postoperative period, the i.v. cannulae were flushed daily with heparin (25 iU/ml) via a sampling line to maintain patency and habituate the rats to experimental handling. In a separate group of animals, rats were surgically prepared for central infusions by stereotaxic implantation with stainless steel tubing i.c.v. guide cannula (22 gauge) terminating 1 mm above the left lateral ventricle (0.8 mm caudal and 1.5 mm lateral to Bregma; coordinates derived from the atlas of Paxinos and Watson (13). Guides were fastened to the skull with microscrews and dental cement. A dummy cannula was inserted into the guide cannula to prevent any blockage during the recovery period and was removed the evening before the experiment to ensure that animals would remain unstressed on the morning of the experiment. Guide and dummy cannulae were obtained from Plastics One (Roanoke, VA, USA). Guide-implanted rats were housed singly following surgery and allowed 5–7 days postoperative recovery before the experiment.

### Experiment 1: Effect of the NOP antagonist, JTC-801, on the HPA axis response to restraint

Twenty-four animals were randomly assigned to four treatment groups. The dose of JTC-801 was selected on the basis that, *in vivo*, when given i.v. at doses 0.01 mg/kg and above, JTC-801 fully attenuates N/OFQ-induced allodynia (8) and is a suitable NOP antagonist for behavioural studies. Because JTC-801 has limited solubility in saline, it was dissolved in dimethylsulphoxide (DMSO). Vehicle (1% DMSO in 0.9% sterile saline) infusion was not associated with any adverse behavioural effects. On experimental day, at time 0, a blood sample (300 μl) was collected via the i.v. cannula. Two of the groups then received i.v. infusions of vehicle (1% DMSO in 0.9% sterile saline, 100 μl total volume) and two groups received i.v. infusions of JTC-801 at 0.05 mg/kg (Tocris, Bristol, UK) dissolved in 1% DMSO/saline vehicle. Rats were subsequently immediately exposed to restraint holders for 60 min (as described above). Control animals remained in their homecage. Blood samples (300 μl) were taken via the i.v. sampling line 30 and 60 min post-drug administration. The sampling line was left attached to animals undergoing restraint but detached from homecage controls so that the latter could move freely. Following 60 min of restraint, rats were returned to their homecage. After each blood sample collection, an equivalent volume of heparinised saline was administered. Plasma samples were prepared by centrifugation before storage at −20 °C.

### Determination of plasma hormone concentrations

Plasma corticosterone (CORT) concentrations were determined as described previously (14) using antiserum supplied by Dr G. Makara (Institute of Experimental Medicine, Budapest, Hungary). The tracer used was [^125^I]-CORT (MP Biomedicals, Cambridge, UK) with a specific activity of 2–3 mCi/mg. The intra-assay coefficient of variation was < 12%.

### Activation of Fos expression following i.c.v. JTC-801

To investigate the central effects of JTC-801 on Fos activation, two groups of male rats were subject to handling and i.c.v. injection (5 μl) of 0.9% sterile saline vehicle or i.c.v. injection of JTC-801 (1 μg/rat in 5 μl of saline). Injections were given between 09.00 h and 10.00 h. Following injections, rats were returned to their homecages and, 90 min post-i.c.v. injection, rats were deeply anaesthetised by an i.p. injection of sodium pentobarbital (40 mg/kg; Euthatal, Rhone Merieux, Toulouse, France). Animals were fixed by transcardial perfusion using 200 ml of 0.1 m phosphate-buffered saline (PBS) followed by 200 ml of 4% paraformaldehyde (PFA) in 0.1 m sodium phosphate (PB) buffer (composition: Na_2_PO_4_; NaH_2_PO_4_.2H_2_O in 1 l dH_2_O, pH 7.4, kept at 4 °C). Brains were then removed, post-fixed overnight in 4% PFA in PB and cryopreserved in 30% sucrose in 0.1 m PB for approximately 72 h. Brains were frozen in liquid nitrogen, trimmed, then mounted on a cryostat chuck for sectioning (Leica, Wetzlar, Germany). Coronal sections (40 μm) of hypothalamus were collected free-floating in 0.2 m PB (0.2 m Na_2_HPO_4_, 0.04 m NaH_2_PO_4_, pH 7.4). Sections were then washed (3 × 5 min) in PBS containing Triton (PBST: 0.1 m PB; 0.9% NaCl; 0.2% Triton-X-100, pH 7.4). Endogenous peroxidase activity was then quenched by incubation in PBST containing 0.3% H_2_O_2_ for 30 min. Following 4 × 10-min washes in PBST, sections were incubated for 1 h at room temperature in a blocking solution comprising PBST and 1.5% normal goat serum to reduce nonspecific binding. Sections were subsequently incubated in rabbit polyclonal anti-cFos antibody (AB5; Calbiochem, Nottingham, UK) at a dilution of 1 : 5000 in PBST at 4 °C for 24 h. Following incubation with primary antibody, sections were washed in PBST (4 × 10 min). Antibody binding was visualised using the Avidin-Biotin Complex (ABC) technique. Sections were incubated with biotin-conjugated secondary antibody, goat anti-rabbit IgG (ABC kit Vectastain Elite PK-6101; Vector Labs, Peterborough, UK) at 1 : 200 in PBST for 1 h at room temperature. Following five further 10-min washes in PBST, sections were incubated in the Avidin-Biotin Complex for 1 h (ABC kit; Vector Labs). Sections were then washed twice in PBST (10 min each), followed by two washes in Tris buffer for 10 min (0.05 m, pH 7.6). Sections were then incubated in substrate solution containing 3,3′-diaminobenzidine peroxidase substrate (DAB substrate kit; Vector Labs) for approximately 5 min, and the reaction stopped in double-distilled water for 5 min. Sections were then dehydrated through ascending alcohol series, cleared with xylene, air-dried and then permanently mounted using DPX mountant and coverslipped. Fos-immunoreactive (-IR) cells were visualised under a Leica light microscope under brightfield illumination (× 40 objective) and bilaterally counted in three sections per parvocellular division of the paraventricular nucleus (pPVN). Omission of primary antibody resulted in absence of staining (negative control). Anatomical verification of subregional staining was determined by reference to the atlas of Paxinos and Watson (13).

### Experiment 2: Stress-induced regulation of ppN/OFQ and NOP receptor mRNA levels in rat forebrain

The timecourse for mRNA expression response was estimated by comparison with psychological stress-induced changes in opioid receptor mRNAs and opioid precursor mRNAs. From the literature, opioidergic systems are acutely regulated by stressors, with the earliest changes in mRNA levels in the midbrain being detected by 30 min post stress and lasting 120 and 360 min following social defeat (15). Yamamoto *et al*. (16) also report single (4 h) and repetitive immobilisation stress (over 2 days) induced increases in mu-opiate receptor mRNA levels in the hypothalamus and midbrain. Therefore, we investigated acute changes in mRNA expression to coincide with the expected response to HPA axis activation following restraint (mRNAs at 2 and 4 h post stress onset). Rats were killed at 2 and 4 h following restraint stress onset and brains retained at −80 °C before analysis. Brains were subsequently dissected according to the method of Glowinski and Iversen (17), as employed in Devine *et al*. (18) and Leggett *et al.,* (4), which was carried out under sterile conditions on pre-chilled Petri dishes. The cerebellum and pons were separated from the rest of the brain and a transverse section made at the level of the optic chiasm that defines the anterior part of the hypothalamus and passes through the anterior commissure. The hypothalamus, hippocampus, medio-dorsal forebrain (midbrain and thalamus) and basal forebrain region (comprising subcortical structures: caudate nucleus, nucleus accumbens, pallidal complex and bed nucleus of stria terminalis, BNST) were dissected and then RNA extracted.

### Isolation of RNA from rat brain

RNA was isolated from rat brain (75 mg samples) by guanidinium thiocyanate-phenol-chloroform extraction in accordance with the method of Chromszynski and Sacchi (19). Brain samples were placed into 750 μl of lysis buffer [4 m guanidinium thiocyanate (denaturing solution), 25 mm sodium citrate, 0.5% sarcosyl, 0.1 m β-mercaptoethanol]. Lysed cell products were transferred to a sterile microcentrifuge tube to which 75 μl of sodium acetate (2 m, pH 4.0), 750 μl of water-saturated phenol and 150 μl of chloroform : isoamyl alcohol (49 : 1) was added. Samples were then shaken for 10 s to form an emulsion and then cooled on ice for 15 min followed by centrifugation at 4 °C for 20 min at 10 000 ***g***. The aqueous upper phase was transferred to a clean microcentrifuge tube and mixed with an equal volume of isopropanol at −20 °C for at least 1 h to allow for RNA precipitation. Following centrifugation at 4 °C for 20 min at 10 000 ***g***, the supernatant was discarded and the RNA pellet resuspended in guanidinium thiocyanate denaturing solution. RNA was reprecipitated by the addition of an equal volume of isopropranol at −20 °C for a further 1 h. Following centrifugation at 4 °C for 10 min at 10 000 ***g***, the RNA pellet was washed in 1 ml of cold 75% ethanol followed by a final centrifugation at 4 °C for 10 min at 10 000 ***g***. The supernatant was discarded and the RNA pellet air-dried and resuspended in RNAse-free H_2_O. RNA concentration was determined by ultraviolet spectrophotometry using GeneQuant Pro (Amersham Biosciences, Little Chalfont, UK) and purity was determined by calculation of A_260/280 nm_ ratios. Total RNA samples (in 20 μl of pure H_2_O) were then stored at −80 °C before RT-PCR.

### RT-PCR for ppN/OFQ and NOP mRNAs

Levels of mRNA for ppN/OFQ, NOP receptor and glyceraldehyde-3-phosphate dehydrogenase (GAPDH, house-keeping gene) were determined by semi-quantitative RT-PCR using the Expand Reverse Transcriptase kit (Roche Applied Science, Burgess Hill, UK). One μg of total RNA and 1 μl of Oligo(dT)_15_ (Roche Applied Science, Burgess Hill, UK) underwent initial denaturation for 10 min at 65 °C in a volume of 10.5 μl of RNAse-free H_2_O and were then immediately cooled on ice. Reverse transcription was then performed at 42 °C for 1 h by addition of 1× Expand Reverse Transcriptase Buffer, 10 mm dithiothreitol (DTT), 1 mm each deoxynucleotide triphosphate (dNTP), 20 U ribonuclease inhibitor (RNAsin; Promega, Southampton, UK) and 50 U Expand Reverse Transcriptase in a total volume of 20 μl. The reaction was terminated by placing sample tubes on ice. Primers for amplification of the cDNA for ppN/OFQ were: sense, 5′-CCCACGGCTGCACCATGAAAATC-3′; antisense, 5′-TTCCTCTCACCTGGCCCTACGAGA-3′ (20) with a predicted PCR amplification product of 840 bp. Primers for the cDNA for NOP receptor were: sense, 5′-ATGGAGTCCCTCTTTCCTGCT-3′; antisense, 5′-ACATGTTGTAGTAGTCGATAGCA-3′ with a predicted PCR amplification product of 399 bp. Primers for the cDNA for GAPDH were: sense, 5′-GAACGGGAAGCTCACTGGCAT-3′; antisense, 5′-GTCCACCACCCTGTTGCTGTTAG-3′ with a predicted PCR amplification product of 308 bp. NOP and GAPDH primer pairs were designed and validated in-house. cDNA aliquots (2 μl) were amplified by PCR in a 25-μl reaction volume by Taq DNA polymerase (0.6 U; ABgene, Epsom, UK) in reaction buffer [75 mm Tris-HCl pH 8.8, 20 mm (NH_4_)_2_SO_4_, 0.1% Tween 20] including 2 mm MgCl_2_, dNTPs (0.2 mm each) and 50 pmol of forward and reverse oligonucleotide primers. Amplification reactions were performed using a thermal cycler (Perkin Elmer, Waltham, MA, USA) and terminated before the end of the exponential phase (preproN/OFQ, 26 cycles; NOP receptor, 26 cycles; GAPDH, 20 cycles). Optimised amplification conditions for PCR involved denaturation at 95 °C for 2 min, followed by the appropriate number of cycles at 95 °C for 20 s, annealing at 55 °C for 45 s, extension at 72 °C for 1 min and completion by final extension at 72 °C for 10 min. Amplified PCR products (15 μl) were mixed with 5 μl of loading buffer [0.5% sodium dodecyl sulphate (SDS), 25 mm ethylenediaminetetraacetic acid, 25% Ficoll, 0.15% bromophenol blue] and resolved on a 2% agarose gel containing 0.1 μg/ml ethidium bromide by electrophoresis. Semi-quantitative analysis of resolved products was achieved by visualisation and scanning densitometric measurement of band intensity using Bio-Rad Quantity One analysis software and the Gel Doc 2000 Gel Documentation System (Bio-Rad, Hercules, CA, USA). The relative band intensity (pixel intensity per band) of PCR product (preproN/OFQ or NOP receptor) was expressed as a ratio against data for the house-keeping gene, GAPDH, determined in the same sample.

### ISHH of ppN/OFQ and NOP mRNAs

To investigate the involvement of ppN/OFQ and NOP genes in stress adaptation, we investigated the effect of repeated restraint using ISHH for brain mRNA expression analysis. For the repeated restraint study, 32 rats were assigned to four separate treatment groups. Group 1 comprised the homecage control group. Group 2 were exposed to restraint (60 min) as above on day 14. Group 3 were exposed to daily restraint from days 1–13 (60 min per day). Group 4 were exposed to daily restraint from days 1–14 (60 min per day). All rats were killed on day 14, 3 h after the last restraint session. Group 3 rats are employed as the control group for Group 4 rats because the latter additionally experience restraint on day 14. Rats were decapitated and their brains were removed, frozen over powdered dry ice and stored at −80 °C. Brain sections were prepared (12 μm) using a cryostat (Leica 1850) and thaw-mounted onto positively charged slides. Tissue sections were stored at −80 °C until required for radioisotopic ISHH assay. Riboprobes were prepared from full-length cDNA inserts cloned into pBluescript SK+ bacterial plasmid vectors [kind gifts from Catherine Mollereau, CNRS Toulouse, France (ppN/OFQ) and Olivier Civelli, University of California, Irvine, USA (NOP)]. cDNA inserts were linearised by digestion with appropriate restriction enzymes: *Bam*H1 for ppN/OFQ antisense; *Xho*1 for ppN/OFQ sense; *Hind*III for NOP antisense and *Not*1 for NOP sense. The integrity of the probes was verified by DNA sequencing. A linearised DNA template was used for riboprobe synthesis by *in vitro* transcription using the MAXIscript kit (Ambion, TX, USA) and ^35^S-αUTP. PpN/OFQ antisense and sense RNA transcripts were generated under the control of T7 RNA polymerase and T3 RNA polymerase, respectively. NOP antisense RNA transcripts were generated using T3 RNA polymerase and NOP sense RNA transcripts using T7 polymerase. Labelled riboprobes were then subjected to alkaline hydrolysis before ISHH assay. Unincorporated nucleotides were removed by salt and ethanol precipitation. In preparation for the ISHH assay, stored slides were defrosted for 10 min at room temperature, and then stacked in Coplin jars for pre-hybridisation washes. Tissue sections underwent fixation in 4% PFA in 0.1 m PBS (5 min), twice rinsed in 0.1 m PBS, acetylated in triethanolamine (1.5%)/acetic anhydride (0.25%) for 10 min, dehydrated in ascending alcohol dips (70–100%), delipidated in chloroform (100%, 5 min), partially rehydrated in ethanol (95%) and finally air-dried. Sections were hybridised with radiolabelled probe (1.5 × 10^6^c.p.m./slide) diluted in hybridisation buffer (composition: 5% dextran sulphate w/v, 1 × Denhardt's solution, 1.2 mm ethylenediamine tetraacetic acid, 50% formamide v/v, 0.1 mg/ml salmon testes DNA, 0.25 mg/ml yeast tRNA, 0.25 mg/ml yeast total RNA, 0.375 m sodium chloride, 24 mm Tris-HCl, pH 7.4, in sterile water), 5m DTT, 10% SDS and 10% sodium thiosulphate. Slides were glass-coverslipped and placed in humidified chambers lined with 3MM paper that was pre-wetted with 50% formamide/4 × SSPE solution. Incubation proceeded at 55 °C for approximately 20 h. The following day, coverslips were removed from the slides by dipping in 4× SSPE/1 mm DTT. Slides were rack-mounted and put through a series of washes of increasing stringency to remove unbound riboprobe. Sections were rinsed four times in 4 × SSPE/1 mm DTT for 5 min at room temperature on a rocker platform, washed in RNAaseA solution for 30 min at 37 °C, washed twice in 2 × SSPE/1 mm DTT for 5 min, then 0.1 × SSPE/1 mm DTT (5 min), 0.1 × SSPE/1 mm DTT (twice for 30 min at 65 °C), 0.1 × SSPE/1 mm DTT at room temperature (5 min), followed by 1-min rinses through an ascending alcohol series (70–100% ethanol) containing 300 mm ammonium acetate. Slides were then air-dried. Once dry, slides were arranged in autoradiography cassettes and apposed to film (Amersham Hyperfilm MP; GE Healthcare, Little Chalfont, UK) alongside ARC-146C ^14^C standards (American Radiolabeled Chemical Inc., St Louis, MO, USA) for 2 weeks followed by film development using Kodak D-19 developer (Kodak, Rochester, NY, USA) and Ilford Hypam rapid fixer (Ilford Photo, Knutsford, UK). Film densitometric analysis was performed using imagej (NIH, Bethesda, MD, USA) with photomicrographs produced using a Leica MZ6 digital camera. Anatomical regions were verified by reference to the atlas of Paxinos and Watson (13). Based on the distribution of mRNAs for ppN/OFQ and NOP receptor (21,22) and the literature concerning their role in specific areas of the brain in the control of stress, we focussed our analysis on the regions: BNST, PVN, hippocampus for NOP mRNA; BNST, amygdala, hippocampus and reticular thalamus for ppNOC mRNA.

### Drugs

JTC-801 [*N*-(4-amino-2-methylquinolin-6-yl)-2-(4-ethylphenoxy-methyl)benzamide monohydrochloride] was obtained from Tocris. JTC-801 was dissolved in a 1% DMSO/0.9% sterile saline before injection.

### Statistical analysis

Plasma CORT levels were analysed for group differences using two-way anova with repeated measures. For RT-PCR studies, Mann–Whitney U-tests were used to compare ratios of the mRNA of interest to GAPDH between groups. ISHH data were analysed using one-way anova with a post-hoc multiple comparisons test. Fos immunohistochemical staining was analysed for group differences using the Mann–Whitney U-test. P < 0.05 was considered statistically significant.

## Results

### Effect of JTC-801 on basal and restraint-stress-induced HPA axis activity and Fos expression in the PVN

**Fig. 1:**
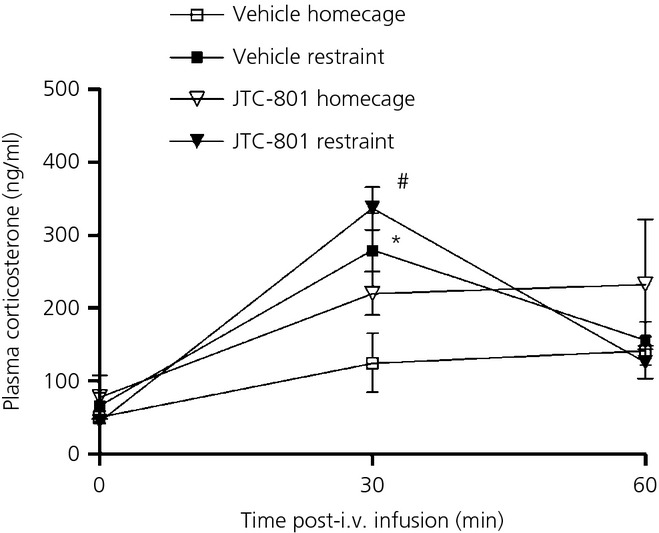
Effect of i.v. infusion (0.1 ml) with vehicle (1% dimethylsulphoxide/0.9% sterile saline) or NOP antagonist, JTC-801 (0.05 mg/kg) dissolved in vehicle on plasma corticosterone levels in response to 60 min of restraint stress in male Sprague–Dawley rats. Nonstress control animals remained in the homecage throughout the experiment. The time 0 blood sample was collected before drug infusions that were immediately followed by placement in restraint holders. Data represent the mean ± SEM plasma corticosterone concentration (ng/ml) (n = 5/6 per group); *P < 0.05, ^#^P < 0.05 versus vehicle homecage group, two-way anova with post-hoc Fisher's protected least significant difference test.

Analysis of differences in plasma CORT levels between each group revealed a significant effect of restraint on plasma CORT across time (F_3,30_ = 24.94, P = 0.0001, n = 5–6), with restraint eliciting a short-lasting but significant increase in plasma CORT at 30 min, followed by a return to baseline (Fig. 1). This finding is concordant with the expected temporal profile of the CORT response to acute restraint. There was a significant treatment × drug interaction, indicating that JTC-801 infusion significantly increased the response of plasma CORT levels in homecage rats compared to vehicle-treated rats (F_3,30_ = 2.56, P = 0.04). By contrast, vehicle infusion in homecage animals was without effect on plasma CORT, with levels significantly lower than the saline-restraint condition at the 30 min time-point (P < 0.05).

**Fig. 2:**
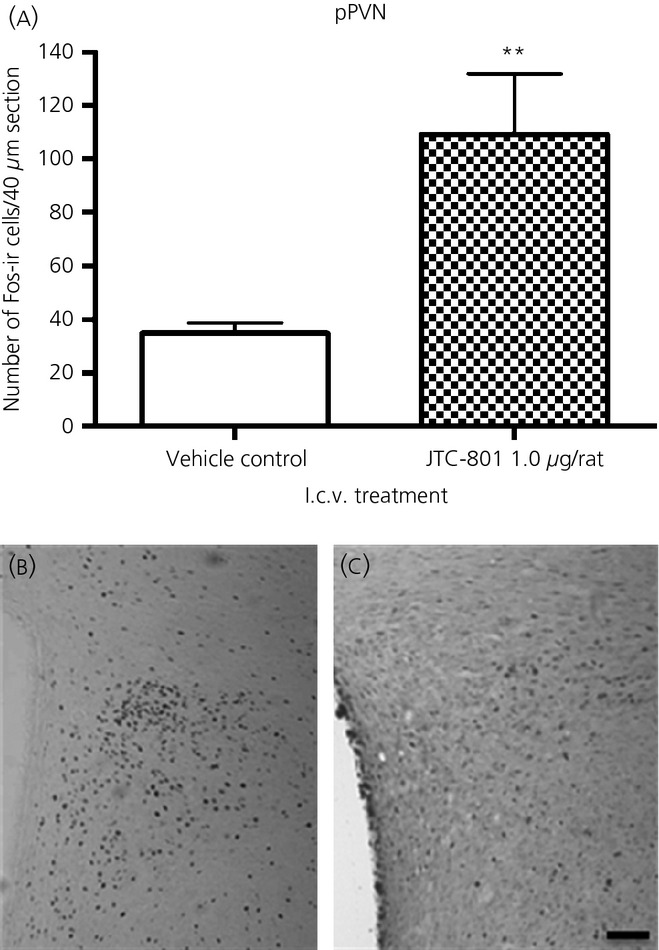
Fos immunoreactivity (-IR) in the parvocellular division of the paraventricular nucleus (pPVN) of male Sprague–Dawley rats 90 min after i.c.v. injection of JTC-801 (1 μg/5 μl sterile saline) or vehicle control (5 μl). Free-floating coronal sections (40 μm) were immunolabelled for Fos-IR in the brain. (a) Quantification of the number of Fos-IR cells in the parvocellular division of the paraventricular nucleus (PVN). Values are expressed as the group mean ± SEM (n = 9 animals). **P < 0.01, vehicle versus JTC-801 group. Representative photomicrographs showing Fos-IR in the PVN of i.c.v. JTC-801-treated rats (b) versus rats treated with i.c.v. vehicle (c). Scale bar = 0.1 mm.

The Mann–Whitney rank sum test revealed that, in male Sprague–Dawley rats, i.c.v. treatment had a significant effect on Fos-IR cells in the pPVN division, with i.c.v. JTC-801 treatment being associated with increased Fos-immunoreactivity compared to i.c.v. vehicle treatment (Mann–Whitney U = 10, P = 0.007). Basal expression of Fos-immunoreactivity was 35 ± 4 cells in vehicle-treated rats versus 109 ± 23 cells in JTC-801 treated rats (n = 9 per group; Fig. 2).

### Effect of acute restraint stress on ppN/OFQ and NOP receptor mRNA expression in rat forebrain

**Fig. 3:**
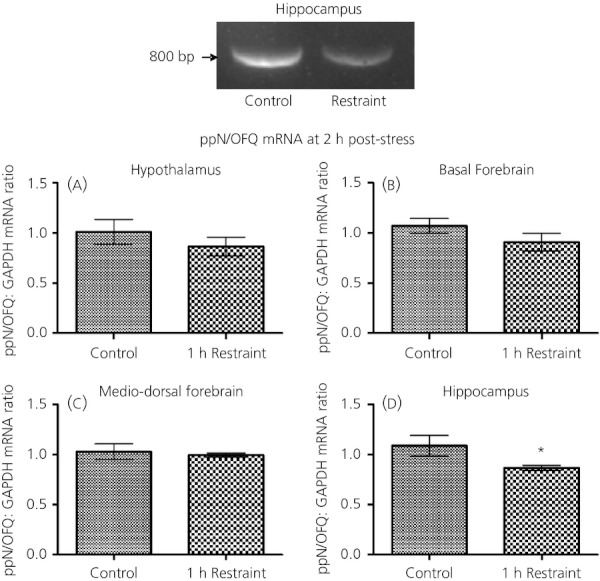
Effect of acute restraint stress (60 min) versus homecage control on ppN/OFQ mRNA expression in the rat (a) hypothalamus, (b) basal forebrain and (c) medio-dorsal forebrain and (d) hippocampus 2 h post-restraint. Data are expressed as ppN/OFQ : GAPDH ratios (mean ± SEM, n = 6). *P < 0.05 versus respective control (Mann–Whitney U-test). The top panel shows a representative image of amplified ppN/OFQ cDNA from hippocampal mRNA in a control versus restraint-stressed animal 2 h following stress onset.

Levels of ppN/OFQ mRNA were examined by RT-PCR in the hypothalamus, basal forebrain, medio-dorsal forebrain and hippocampus from the rat. Differences in hippocampal ppN/OFQ mRNA were apparent between treatment groups at 2 h post-stress onset (Fig. 3). Analysis revealed a significant difference in ppN/OFQ : GAPDH ratios in the hippocampus (1.04 versus 0.87 between control and restraint groups, respectively; Mann–Whitney U = 3.000, P = 0.028; Fig. 3d) representing a 22% decrease in ppN/OFQ mRNA in this region. However, the apparent trends towards a decreases in ppN/OFQ mRNA in the hypothalamus (Mann–Whitney U = 9.000, P *=* 0.283; Fig. 3a) and basal forebrain (Mann–Whitney U = 8.000, P = 0.214; Fig. 3b) did not reach statistical significance. Levels of ppN/OFQ mRNA in the medio-dorsal forebrain in rats subjected to restraint stress were equivalent to controls (Mann–Whitney U = 16.000, P = 1.000; Fig. 3c) (n = 6 per group).

**Fig. 4:**
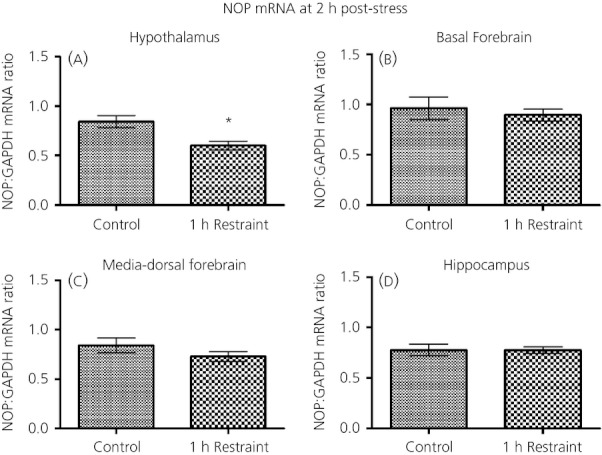
Effect of acute restraint stress (60 min) versus homecage control on NOP mRNA expression in the rat (a) hypothalamus, (b) basal forebrain, (c) medio-dorsal forebrain and (d) hippocampus 2 h post-restraint. Data are expressed as NOP : GAPDH ratios (mean ± SEM, n = 6). *P < 0.05 versus respective control (Mann–Whitney U-test).

In addition to ppN/OFQ mRNA, NOP receptor mRNA levels in the same samples were also assessed. There was a decrease in NOP : GAPDH ratios between stressed animals and controls in the hypothalamus (0.84 versus 0.57, Mann–Whitney U = 3.00, P = 0.028; Fig. 4a) reflecting a 32% decrease in mRNA in this region. At 2 h post-stress onset, levels of NOP mRNA in the basal forebrain (Mann–Whitney U = 14.00, P = 0.808; Fig. 4b), medio-dorsal forebrain (Mann–Whitney U = 8.000, P = 0.214; Fig. 4c) and hippocampus (Mann–Whitney U = 16.000, P = 1.0000; Fig. 4d) in rats in the restraint stress group did not significantly differ from those in the control group (n = 6 per group).

**Fig. 5:**
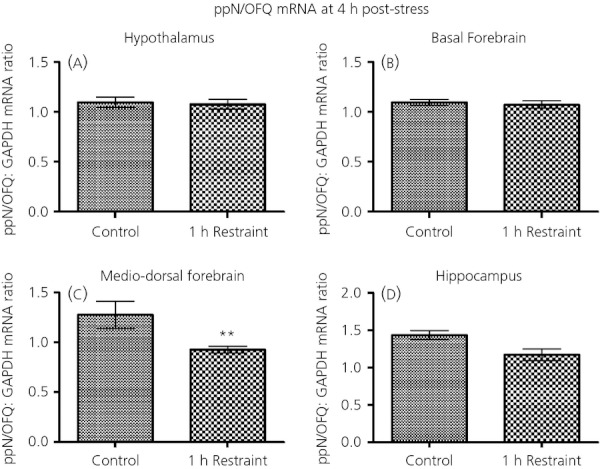
Effect of acute restraint stress (60 min) versus homecage control on ppN/OFQ mRNA expression in the rat (a) hypothalamus, (b) basal forebrain, (c) medio-dorsal forebrain and (d) hippocampus 4 h post-restraint. Data are expressed as ppN/OFQ : GAPDH ratios (mean ± SEM, n = 6). **P < 0.01 versus respective control (Mann–Whitney U*-*test).

In rats killed at 4 h following the onset of stress, the profile of ppN/OFQ mRNA expression differed from those observed at 2 h post-stress onset. As shown in Fig. 5, ppN/OFQ : GAPDH ratios in the medio-dorsal forebrain of rats in the restraint stress condition were significantly reduced relative to controls (1.26 versus 0.94, Mann–Whitney U = 1.00, P = 0.008; Fig. 5c), suggesting a 26% reduction in ppN/OFQ mRNA. Expression of ppN/OFQ mRNA in the hypothalamus (Mann–Whitney U = 12.00, P = 0.788; Fig. 5a) and basal forebrain (Mann–Whitney U = 12.00, P = 0.570; Fig. 5b) was equivalent to controls. Although a trend towards a decrease in hippocampal ppN/OFQ mRNA was observed, it did not reach statistical significance (Mann–Whitney U = 5.00, P = 0.073; Fig. 5d) (n = 6 per group).

**Fig. 6:**
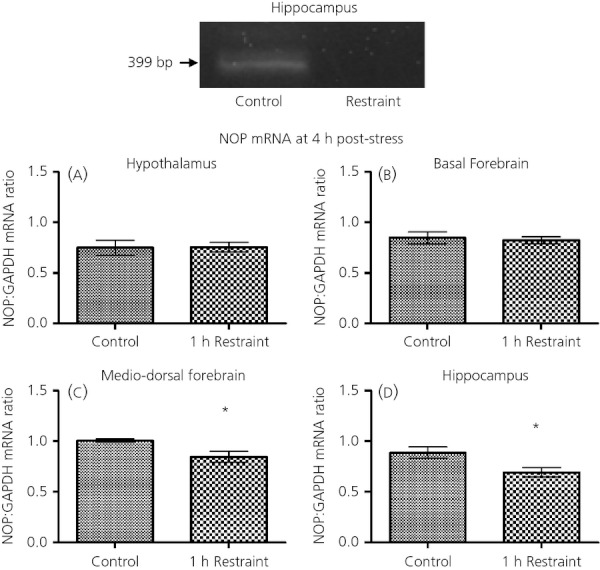
Effect of acute restraint stress (60 min) versus homecage control on NOP mRNA expression in the rat (a) hypothalamus, (b) basal forebrain, (c) medio-dorsal forebrain and (d) hippocampus 4 h post-restraint. Data are expressed as NOP : GAPDH ratios (mean ± SEM, n = 6). *P < 0.05 versus respective control (Mann–Whitney U-test). The top panel shows a representative image of amplified NOP cDNA from hippocampal mRNA in a control versus restraint-stressed animal 4 h following stress onset.

Hypothalamic NOP receptor mRNA expression, which had been reduced in animals subjected to restraint stress at 2 h post-stress onset, was at control levels at 4 h post-stress onset (Mann–Whitney U = 12.00, P *=* 0.79; Fig. 6a). Similarly, there was no difference in NOP mRNA levels in control versus restraint animals in the basal forebrain (Mann–Whitney U = 12.00, P = 0.57; Fig. 6b). However, NOP : GAPDH ratios in the medio-dorsal forebrain (1.01 versus 0.87, Mann–Whitney U = 3.00, P = 0.0283; Fig. 6c) and hippocampus (0.84 versus 0.71, Mann–Whitney U = 4.00, P = 0.049; Fig. 6d), which had been equivalent to controls in the group sacrificed 2 h post-stress, were significantly reduced 4 h after the onset of restraint stress, suggesting a decrease in NOP receptor mRNA in these regions.

### Effect of repeated restraint stress on ppN/OFQ and NOP receptor mRNA expression in rat brain

**Fig. 7:**
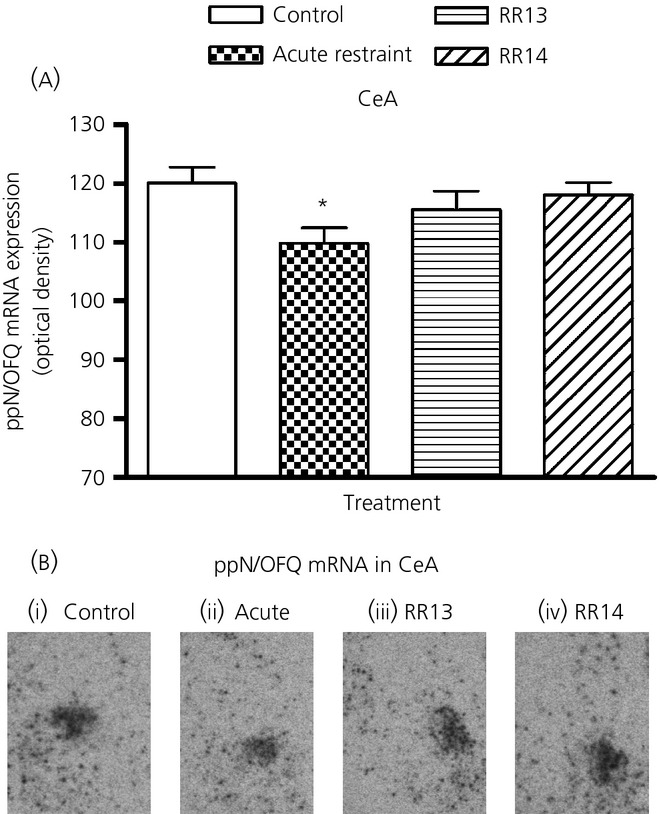
ppN/OFQ mRNA expression in the central amygdala (CeA) 3 h following onset of restraint stress. (a) Rats were either homecage controls, or subjected to acute restraint (60 min on day 14), repeated restraint (60 min per day for 14 days, killed on day 14; RR14) or repeated restraint (60 min per day for 13 days, killed on day 14; RR13). Data represent the mean ± SEM optical density (n = 6–7); *P < 0.05 versus all other groups, one-way anova and post-hoc Fisher's protected least significant difference test. (b) Expression of ppN/OFQ mRNA in a coronal section of rat brain. Images represent bright-field film photomicrographs of CeA hybridised to ^35^S-labelled antisense riboprobe in (i) control, (ii) Acute restraint, (iii) Repeated restraint for 13 days and (iv) Repeated restraint for 14 days.

**Fig. 8:**
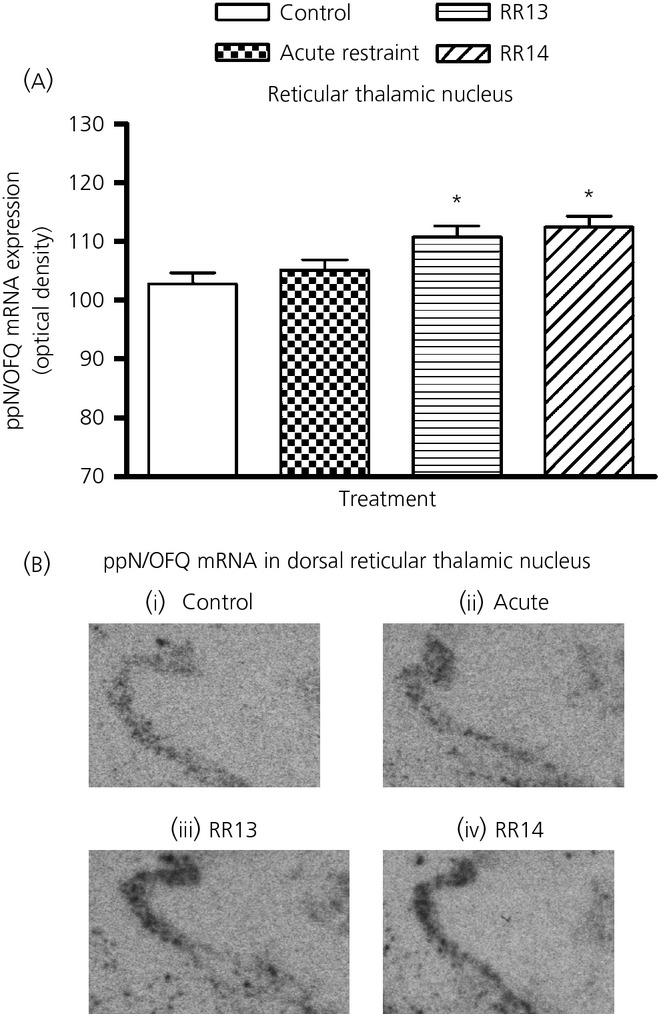
ppN/OFQ mRNA expression in the bed nucleus of stria terminalis (BNST) 3 h following start of restraint stress (a). Rats were either homecage controls, or subjected to acute restraint (60 min per day for 14 days, killed on day 14; RR14) or repeated restraint (60 min per day for 13 days, killed on day 14; RR13). Data represent the mean ± SEM optical density (n = 4–8); *P < 0.05 versus control and acute stress groups, one-way anova and post-hoc Fisher's protected least significant difference test. (b) Expression of ppN/OFQ mRNA in coronal sections of the rat brain. Images represent bright-field film photomicrographs BNST hybridised to ^35^S-labelled antisense riboprobe in (i) control, (ii) acute restraint, (iii) repeated restraint for 13 days and (iv) repeated restraint for 14 days. AC, anterior commissure.

**Fig. 9:**
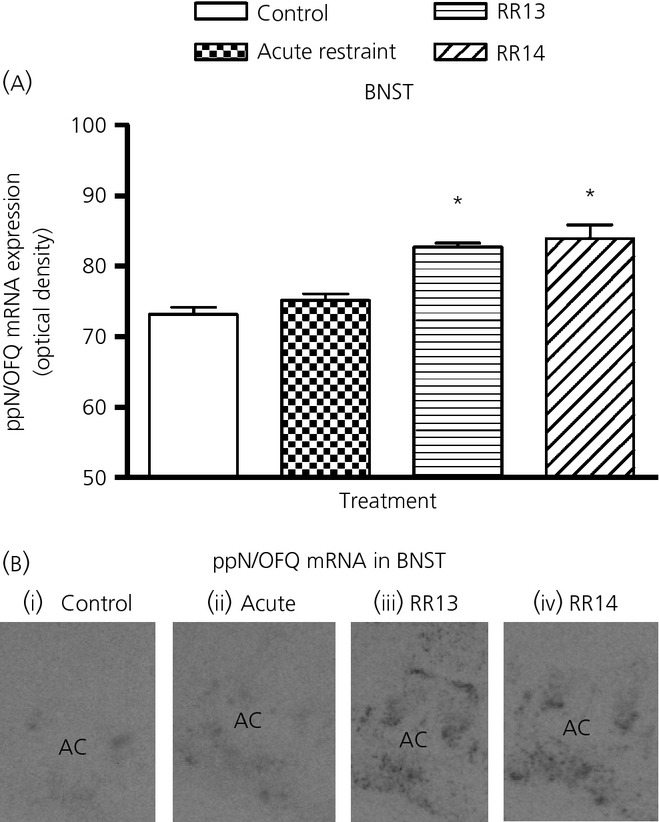
ppN/OFQ mRNA expression in the reticular thalamic nucleus (RtN) 3 h following start of restraint stress. (a) Rats were either homecage controls, or subjected to acute restraint (60 min on day 14), repeated restraint (60 min per day for 14 days, killed on day 14; RR14) or repeated restraint (60 min per day for 13 days, killed on day 14; RR13). Data represent the mean ± SEM optical density (n = 5–7); *P < 0.05 versus control and acute stress groups, one-way anova and post-hoc Fisher's protected least significant difference test. (b) Expression of preproN/OFQ mRNA in a coronal section of rat brain. Images represent bright-field film photomicrographs of RtN hybridised to ^35^S-labelled antisense riboprobe in (i) control, (ii) acute restraint, (iii) repeated restraint for 13 days and (iv) repeated restraint for 14 days.

**Fig. 10:**
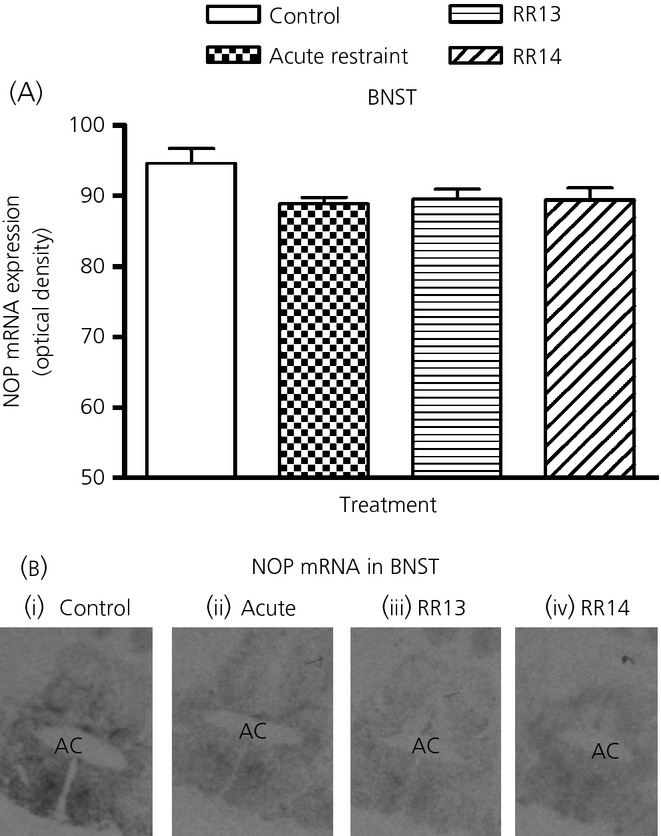
NOP mRNA expression in the bed nucleus of stria terminalis (BNST) 3 h following start of restraint stress (a). Rats were either homecage controls, or subjected to acute restraint (60 min on day 14), repeated restraint (60 min per day for 14 days, killed on day 14; RR14) or repeated restraint (60 min per day for 13 days, killed on day 14; RR13). There was a strong trend suggesting an acute and repeated stress-induced reduction in NOP mRNA (P = 0.053, one-way anova). Data represent the mean ± SEM optical density (n = 5–7). (b) Expression of NOP mRNA in a coronal section of rat brain. Images represent bright-field film photomicrographs of BNST hybridised to ^35^S-labelled antisense riboprobe. AC, anterior commissure.

PpN/OFQ and NOP mRNA distribution in brain regions from male Sprague–Dawley rats was consistent with previous reports using ISHH analysis (21–24,33). PpN/OFQ mRNA was scattered throughout the forebrain, including the cerebral cortex, hypothalamus, hippocampus, bed nucleus, amygdala and reticular thalamic nucleus. NOP mRNA was distributed throughout the forebrain, with particularly high levels in the cerebral cortex, hippocampus, amygdala, bed nucleus and hypothalamus. Semi-quantitative ISHH analysis revealed site-specific differences in mRNA expression between treatment groups, in particular for ppN/OFQ mRNA levels. ISHH did not reveal major acute restraint-induced changes in ppN/OFQ mRNA expression, with the exception of reduced transcript levels in the central nucleus of the amygdala (CeA) (F_3,23_ = 3.26, P = 0.044; Fig. 7). Repeated restraint-stressed rats (RR13 and RR14) were not significantly different to controls with respect of ppN/OFQ mRNA levels in CeA. There were significant differences between the mean densities of the treatment groups in BNST ppN/OFQ mRNA expression (F_3,23_ = 25.34, P = 0.0001; Fig. 8). PpN/OFQ signal was significantly increased in the BNST in repeated restraint-stressed rats, either stressed for 13 or 14 days, compared to homecage control and acute restraint groups (P < 0.05). Furthermore, although acute restraint had no effect on ppN/OFQ mRNA in the reticular thalamus, repeated restraint stress (RR13 and RR14 groups) significantly increased the expression of precursor transcript in this region (F_3,23_ = 3.85, P = 0.045; Fig. 9). By contrast to the effects of restraint stress on the peptide precursor mRNA, ISHH revealed few significant differences in density between treatment groups for limbic NOP mRNA expression. Although there was a strong trend towards reduced NOP mRNA density following acute and repeated restraint in the BNST (F_3,23_ = 3.035, P = 0.053, not significant; Fig. 10), there was no effect of restraint on NOP mRNA expression in other limbic or hypothalamic regions, including the pPVN (F_3,25_ = 1.022, P = 0.402, not significant; Fig. 1).

**Fig. 11:**
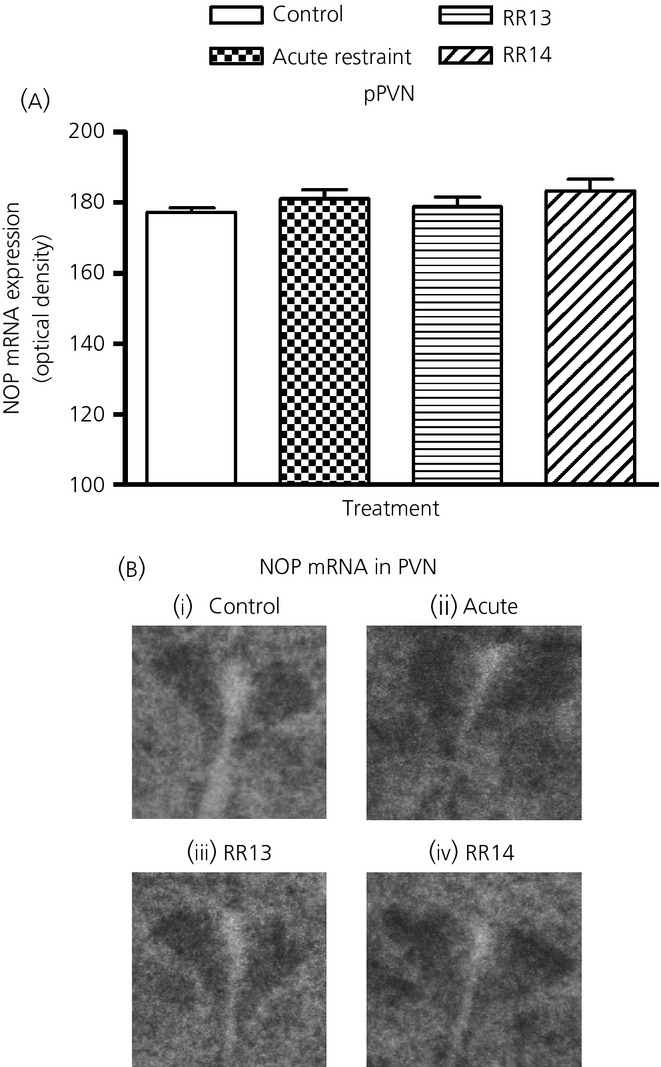
NOP mRNA expression in the parvocellular region of the hypothalamic paraventricular nucleus (pPVN) 3 h following start of restraint stress (a). Rats were either homecage controls, or subjected to acute restraint (60 min on day 14), repeated restraint (60 min per day for 14 days, killed on day 14; RR14) or repeated restraint (60 min per day for 13 days, killed on day 14; RR13). Data represent the mean ± SEM optical density (n = 6–7). PVN NOP mRNA was not altered by acute or repeated restraint stress at 3 h. (b) Expression of NOP mRNA in a coronal section of rat brain. Images represent bright-field film photomicrographs of PVN hybridised to ^35^S-labelled antisense riboprobe.

## Discussion

N/OFQergic neurones are involved in many different functions, including pain, anxiety, endocrine and autonomic regulation. With respect to the neuroendocrine role of N/OFQ, we aimed to further investigate the endogenous role of NOP receptors in the regulation of basal and stress-induced HPA axis activity and also the adaptation of the central N/OFQ system to psychological stress. We first tested the effect of a putative synthetic NOP antagonist JTC-801 on the plasma CORT response to restraint. In our previous research, prolongation of the restraint-stress-induced HPA axis response was seen with the peptidergic NOP antagonist UFP-101 (3). In the present study, a significant effect of restraint stress on HPA axis activation was observed with raised plasma CORT at 30 min post-stress onset, with plasma CORT levels being indistinguishable from controls at 60 min post-stress. Although there was no main effect of JTC-801 on the HPA axis response to acutely restrained animals, there was a significantly enhanced HPA axis response to JTC-801 infusion in homecage (unstressed) rats. This finding suggests that, under basal conditions, JTC-801 is able to elicit a modest activation of the HPA axis, in a manner similar to that seen previously using the central administration of N/OFQ peptide (1,2). In agreement with our observation regarding CORT release, and additionally to determine the site of action of the drug, JTC-801 delivered by i.c.v. injection significantly increased Fos-like immunoreactivity in the medial pPVN compared to vehicle-treated control rats. This Fos activation was consistent with the distribution of CRF hypophysiotrophic neurones (25,26), and supports the findings showing i.v. JTC-801 induced stimulation of basal HPA axis activity. The fact that i.c.v. JTC-801 elicits Fos activation in the pPVN also implies that i.v. JTC-801-induced release of CORT is mediated centrally at the level of the hypophysiotrophic pPVN neurones. These findings suggest that JTC-801 may elicit a pharmacological stress response, with implications for understanding the role of endogenous N/OFQ action *in vivo*. Because N/OFQ peptide i.c.v. also elicits an increase in Fos expression in pPVN in a similar distribution pattern to JTC-801 (27), we suggest that JTC-801 itself may exert some partial agonist-like activity at NOP receptors under the conditions employed in the present study.

Following i.c.v. delivery, JTC-801 could bind to NOP receptors in structures adjacent to the third ventricle or influence the complex neuronal circuitry impinging on hypophysiotrophic CRF neurones. Although the actions of the nonpeptidergic NOP antagonist JTC-801 suggest that endogenous NOP tonically regulates the HPA axis under basal conditions, there was no evidence for JTC-801-induced modulation of restraint-induced HPA axis activity. This finding is in contrast to our recent data showing that i.c.v. administration of the peptidic NOP antagonist, UFP-101, results in an enhanced and prolonged HPA axis response to acute restraint stress, with no effect on basal HPA axis activity *per se* (2,3). Thus, the action of JTC-801 in the HPA axis appears to be distinct from that of peptidic antagonist, UFP-101, raising the possibility that JTC-801 is not acting as a pure NOP antagonist. Both JTC-801 and UFP-101 have been suggested to have complex pharmacological profiles under certain conditions, including inverse agonism (JTC-801) and partial agonist activity (UFP-101) (28). It is also possible that JTC-801 could interact with a different site on the NOP receptor to the N/OFQ binding site, in effect acting as an allosteric regulator (7,29). It is apparent that the potential for differential pharmacological actions *in vivo* must be a consideration for all NOP antagonists. Indeed, NOP receptor ligands have demonstrated mixed pharmacological profiles with respect of other functional responses; for example, regulation of cardiovascular and renal control in conscious animals (30).

Previous evidence has suggested that JTC-801 is able to block endogenous N/OFQ function in a manner that produces reliable pharmacological antagonism. Intravenous JTC-801 at doses of 0.01 mg/kg and above antagonises N/OFQ-induced hyperalgesia (8,31) and has anti-nociceptive effects in acute pain models in mice when administered alone (8). In rats, JTC-801 given orally in food alleviates heat-evoked hyperalgesia in osteoporosis induced by chronic constriction injury (CCI) (32) and JTC-801 (1 mg/kg i.p) functionally antagonises cannabinoid-induced hypothermia in rats (12). The present study is the first to employ JTC-801 with respect to HPA axis regulation. However, there are a number of considerations that may have influenced the effect of JTC-801 in our studies, including pharmacokinetics of this NOP antagonist *in vivo*, in addition to some debate concerning the selectivity profile of JTC-801 at the brain NOP receptor. Differences in NOP antagonist effects on the HPA axis may relate to dose–response characteristics, differences in binding and receptor-ligand interactions between peptidic and nonpeptidic compounds or the route of administration. We found evidence for HPA axis stimulation in both i.v. and i.c.v. JTC-801-injected rats. The JTC-801 dose was based on previous reports of actions *in vivo* in mice and rats. JTC-801 has been administered i.v. in mouse models, whereas, in rats, JTC-801 has been given by the oral or i.p. route. It is possible that the dose administered i.v. in the present study was suboptimal for full blockade of central NOP receptors in rats; however, extensive dose–response studies would be required to examine this possibility with respect to HPA axis function. As stated earlier, the dissimilarity in our data between the actions of UFP-101 versus JTC-801 on stress-induced CORT responses may also suggest that the latter compound is not a purely competitive NOP antagonist. We suggest that the outcome of neuropharmacological studies employing NOP ligands *in vivo* may also be strictly dependent on experimental conditions.

Brain N/OFQergic neurones are stress responsive (4,18,24,33); however, the mechanisms of action of N/OFQ responsive pathways that underpin acute or chronic stress adaptation are poorly understood and cannot be determined solely using pharmacological studies. A more beneficial approach to understanding adaptive responses in N/OFQ neurones in stress is to monitor directly psychological stress-induced changes in the expression of the N/OFQ or NOP system. We investigated the regulation of N/OFQ system gene transcript expression in the limbic forebrain of restraint-stressed rats. We adopted two techniques: (i) RT-PCR to assess the effect of single restraint exposure on N/OFQ precursor and NOP mRNA levels in brain homogenates and (ii) repeated restraint-stress-induced changes in gene transcripts measured using ISHH in coronal brain sections. RT-PCR analysis found significant acute restraint-induced reductions in gene expression in the medio-dorsal forebrain region (encompassing thalamus and midbrain) and hippocampus, without a change in other regions, including the hypothalamus. Specifically, N/OFQ precursor mRNA was reduced 2 h following acute restraint in the hippocampus (returning to control level at 4 h) and reduced at 4 h in the medio-dorsal forebrain. PpN/OFQ transcript was also reduced in the medio-dorsal forebrain and hippocampus 4 h following acute stress-onset. Previous research has found that acute stress (30 min of restraint) has little effect on N/OFQ peptide content in the spinal cord and most brain regions, except the basal forebrain where significant reductions (25–30%) were found immediately post-stress (18). Although RT-PCR analysis found quite spatially-restricted changes in ppN/OFQ mRNA, the magnitude of the acute stress-induced change in transcript levels was consistent with the decrease in peptide content reported by Devine *et al*. (18). Furthermore, Devine *et al*. (18) found a clear trend towards a reduction in N/OFQ peptide content in the midbrain following acute psychological stress, and our data suggest that this could be associated with a reduction in N/OFQ gene expression. Reductions in the expression of both N/OFQ precursor and NOP receptor mRNAs in the medio-dorsal forebrain implicate changes in stress-responsive N/OFQergic populations, including midbrain monoaminergic cell groups that highly express N/OFQ precursor and/or NOP mRNA (21,22), and are relevant to affective behaviour.

Regional changes in stress responsiveness of N/OFQergic neuronal populations may be related to essential differences in the type and duration of acute stress paradigms. Interestingly, RT-PCR analysis also revealed that acute restraint (60 min) significantly reduced NOP mRNA synthesis in the hypothalamus at 2 h (returning to control at 4 h), despite no change in ppN/OFQ mRNA level. Thus, it appears that acute restraint regulates the N/OFQergic system of the hypothalamus primarily via changes in NOP receptor gene expression. Given that restraint stress evoked somewhat modest detectable changes in mRNAs by RT-PCR, we concluded that this technique had limitations in sensitivity for monitoring stress-induced changes in mRNA transcripts. However, in another study using a different cohort of rats, ISHH analysis of the medial parvocellular PVN did not find any significant changes in NOP mRNA expression 3 h post-stress. ISHH was undertaken in an attempt to provide improved spatial information concerning subregional changes in gene expression following stress. ISHH analysis identified significant acute stress-induced changes in N/OFQ system gene transcript levels, although the reductions in ppN/OFQ mRNA were localised to the CeA. This may be significant because the CeA is the major output nucleus of the amygdala, a complex critically important for integration of stress and anxiety behaviours, and the CeA is implicated in fear, autonomic and endocrine responses to stress. Acute restraint stress-induced reduction in CeA ppN/OFQ mRNA is consistent with stress-induced regulation of limbic N/OFQergic neurones and potential down-regulation of ppN/OFQ transcription in response to elevated glucocorticoid levels.

Although we did not find acute restraint-induced changes in hippocampal ppN/OFQ mRNA using ISHH, as we did using RT-PCR, this may be a consequence of variation in stress-responsiveness between different cohorts of rats. An alternative explanation for this discrepancy is methodological approach, with RT-PCR involving amplification of mRNA extracted from whole hippocampal homogenates versus mRNA detection in cryosections of hippocampal formation by ISHH that enable the laminar structure to be visualised. The disadvantage of ISHH is that, despite the benefit of improved spatial resolution, signal intensity may be reduced and thus stress-induced effects may be below the limit of detection for ISHH. Brain samples were obtained 2 and 4 h post-stress for RT-PCR analysis and 3 h post-stress for ISHH; therefore, temporal differences may also have contributed to the variation in the mRNA response observed in the two experiments. Each technique has inherent advantages and disadvantages; therefore, this emphasises the importance of using more than one technique to investigate gene expression responses to psychological stressors. The development of specific probes to detect changes in heteronuclear RNA for ppN/OFQ and NOP, in addition to real-time PCR analysis for mRNA, would also be needed to facilitate detection and quantification of rapid, site-specific changes in gene expression following stress exposure. Such an approach would provide a more sensitive methodology and reveal subtle changes in expression levels over time.

Previous evidence from *in vivo* studies also suggests that the role of N/OFQ in the hippocampus may not be critical for anxiety regulation and adaptive fear, given that intra-hippocampal injection of N/OFQ or NOP antagonists do not affect anxiety behaviour (34). Intra-hippocampal injection of NOP antagonist, UFP-101, however, elicits antidepressant behaviour (35) and intra-hippocampal N/OFQ inhibits learning and memory (34); therefore, there is some evidence to suggest that the functional role of the hippocampal N/OFQ system is highly context specific and that other limbic structures may be essential for adaptive stress responses. This may be relevant given the relatively modest changes in hippocampal transcript expression that we detected following restraint stress.

The CeA is of interest given that it is reciprocally connected to the BNST, a major limbic region implicated in the integration of HPA axis responses and stress behaviour. Interestingly, N/OFQ infusion intra-BNST attenuates CRF-induced anxiety in the elevated plus maze test in rats (36) and antagonises CRF-induced anorexia in rats. Therefore, in the context of adaptive stress responses, our present observation that repeated restraint stress for either 13 or 14 consecutive days resulted in up-regulation of ppN/OFQ mRNA expression in the BNST as shown by ISHH, without a marked change in NOP mRNA expression, is significant and supports the role of BNST N/OFQ-expressing neurones in homeostatic responses to repetitive stress. Using RT-PCR analysis, we did not detect an effect of acute restraint on either N/OFQ precursor or NOP mRNA expression level 2 or 4 h following stress onset in the basal forebrain region encompassing the BNST, a region previously shown to be responsive to an immunological stressor (4). It is intriguing that BNST ppN/OFQ mRNA was not changed by acute restraint, yet was robustly up-regulated following repeated restraint exposure. Devine *et al*., (18) found moderate reductions in basal forebrain N/OFQ peptide content in association with acute restraint stress but resistance to changes with chronic variable stress. Control of N/OFQergic neurones in terms of rate of N/OFQ biosynthesis versus bioactive peptide levels may be independently regulated by acute and chronic stressors. In response to repeated restraint stress, increased stimulation of the BNST (possibly through the development of imbalance between amygdala–BNST inputs) could account for a localised increase in BNST N/OFQ mRNA expression, possibly in response to increased peptide release associated with prolonged stress. There was also a clear trend showing that acute and chronic restraint were associated with decreased NOP mRNA in the BNST when measured using ISHH (P = 0.053). This emphasises the benefits of the ISHH approach that permits more precise localisation of limbic mRNA transcripts because discrete changes in subregional expression may be missed if RT-PCR is employed exclusively. In terms of an interpretation of the BNST mRNA expression, enhanced BNST N/OFQ release (and secondary increase in ppN/OFQ mRNA) could result in decreased NOP mRNA expression as a compensatory response to resist ongoing stress-induced activation of N/OFQ neurones. Given that there is currently very little literature available concerning the transcriptional rates of ppN/OFQ or NOP genes, or indeed the molecular mechanisms underpinning their regulation, it is difficult to infer the significance of changes in steady-state mRNA expression or mRNA turnover in relation to peptide levels.

PpN/OFQ mRNA was also significantly increased in the dorsal reticular thalamic nucleus of repeated restraint rats, without a change in acute stress. N/OFQ mRNA expression is particularly dense in this nucleus, indicating a potential role for N/OFQ in the modulation of afferent inputs arising in the ventral striatum, brainstem, substantia nigra and globus pallidus. Reticular thalamic neurones are all GABAergic, thereby highlighting a role for N/OFQ in the regulation of inhibitory GABAergic control and integration of the dorsal thalamus. The activity of inhibitory neurones of the reticular thalamus is critical for behavioural state and selective attention, and recent studies implicate the dorsal region in anxiety and fear-related behaviour (37). 6-Hydroxydopamine lesions of the dorsal reticular thalamus attenuate anxiety in the elevated plus maze and shock-probe burying tasks in rats, without changes in motor activity (37). To our knowledge, this is the first report demonstrating the involvement of the dorsal reticular thalamus in chronic stress adaptation. Although delta opioid receptor expression is decreased in the reticular thalamus associated with monoarthritis (38) and somatostatin mRNA in mouse reticular thalamus is up-regulated in the weaver mouse mutant, a model characterised by deficiency in brain dopamine (39), no studies on stress-induced responses of reticular thalamus have been reported. Repeated restraint in rats is known to elicit anxiety; thus, it is possible that increased ppN/OFQ mRNA in the reticular thalamus correlates with anxiety state. Although evidence suggests that N/OFQ itself is anxiolytic (40–42), it is possible that chronic stress increases N/OFQ transcription in the reticular thalamus. Increased N/OFQ neurotransmission may attenuate GABA function in the dorsal thalamic nucleus, manifesting as elevated anxiety. Indeed, recent research suggests that N/OFQ may be anxiogenic under certain conditions (43). Clearly, further research is required to dtermine the role of N/OFQ in dorsal reticular thalamic functions relevant to behaviour and chronic stress.

This is the first study to investigate the effects of acute and repeated restraint stress on N/OFQ system gene expression. At present, little is known about ppN/OFQ or NOP gene transcription rates and gene regulation, or indeed ppN/OFQ or NOP mRNA turnover. By using two methods suitable for investigating gene transcripts, we have provided insight into steady-state mRNA expression following stress. Stressors may exert a considerable effect on the N/OFQ peptide system, influencing peptide levels, receptor availability and tonic peptide regulation of systems, including the HPA axis. Our present findings strongly suggest that restraint elicits notable adaptations in gene expression that support a stress compensatory role for the endogenous N/OFQ system, in particular in the BNST and associated limbic network following HPA axis activation. However, it is important to consider that, because the ppN/OFQ gene also encodes other bioactive peptides in addition to N/OFQ, such as nociceptin II and nocistatin (44), it is evident that changes in the functional regulation of each of these products in turn, and in a site-specific manner, could contribute to the complex adaptive responses to restraint. Interestingly, we have previously found marked glucocorticoid-induced suppression of N/OFQ function in BNST *in vitro* (45). Taken together, the mRNA results are suggestive of specific, stress-induced changes in ppN/OFQ transcript in particular, with NOP mRNA expression apparently being more resistant to stress with relatively stable expression. Multiple, functional glucocorticoid response elements (GREs) have been identified in the human N/OFQ precursor gene (46); thus, it is possible that acute and chronic restraint stress-induced fluctuations in brain glucocorticoids could directly impact central N/OFQ precursor gene transcription. Furthermore, mRNA turnover for G-protein-coupled receptors can be regulated by glucocorticoids and, given that five GREs in NOP have been identified (47), potentially, such steroids could directly regulate NOP gene transcription. Our findings suggest that acute restraint stress results in a reduction of N/OFQ gene expression. This could involve a direct glucocorticoid-induced decrease in N/OFQ biosynthesis or a decrease in stability of the ppN/OFQ transcript leading to increased transcript degradation. Further work is required to establish whether ppN/OFQ mRNA is indeed inhibited by peripheral circulating glucocorticoids.

It is interesting to compare our ISHH data with those of Green and Devine (24) who reported the effects of acute and repeated social defeat on N/OFQ system expression in rats. Acute and repeated social defeat had no effect on ppN/OFQ mRNA in any region measured. By contrast, acute social defeat was found to increase NOP mRNA expression in the CeA and basomedial amygdala, with both acute and repeated defeat stress causing up-regulation of NOP mRNA in the PVN of rats. These results contrast with the effects of acute and repeated restraint for which we reported few changes in NOP mRNA detected using ISHH analysis, although RT-PCR did suggest that hippocampal and medio-dorsal forebrain NOP mRNA was reduced following acute stress. Clearly, restraint and social defeat have differential impacts on NOP mRNA expression and this must relate to the neurocircuits activated in response to these psychological challenges. Our results and those of Green and Devine (24) do suggest that psychological stressors evoke fairly modest changes in N/OFQ system gene expression overall; however, the ability of chronic stressors to exert significant change over gene regulation implies that persistent changes in the dynamics of N/OFQergic neuronal regulation contribute to habituation to stress.
